# Successful resolution of methicillin-sensitive *Staphylococcus aureus* bacteraemia complicated by infective endocarditis in a patient with malignancy: a case report of effective combined intravenous nafcillin and ceftaroline therapy

**DOI:** 10.1093/ehjcr/ytag060

**Published:** 2026-02-09

**Authors:** Joshua Modrick, Michelle Malik, Mohammad Azfar Bilal, Sajith Matthews

**Affiliations:** Department of Internal Medicine, Wayne State University School of Medicine, Detroit Medical Center, 4201 St.Antoine, University Health Center Suite 9C, Detroit, MI 48201, USA; Department of Internal Medicine, Wayne State University School of Medicine, Detroit Medical Center, 4201 St.Antoine, University Health Center Suite 9C, Detroit, MI 48201, USA; Department of Cardiology, Wayne State University School of Medicine, Detroit Medical Center, 3990 John R., 4 Hudson, Detroit, MI 48201, USA; Department of Internal Medicine, Wayne State University School of Medicine, Detroit Medical Center, 4201 St.Antoine, University Health Center Suite 5C, Detroit, MI 48201, USA

**Keywords:** Case report, Infective endocarditis, MSSA bacteraemia, Ceftaroline, Nafcillin, Cancer

## Abstract

**Background:**

Infective endocarditis (IE) is a life-threatening complication of bacteraemia, with a high mortality rate especially in immunocompromised individuals. Cancer patients receiving chemotherapy through implanted venous access devices are at an elevated risk of healthcare-associated bacteraemia. While surgery is often indicated for persistent bacteraemia or embolic complications, it may be contraindicated in patients with significant comorbidities. Combination beta-lactam therapy, particularly with nafcillin and ceftaroline, has shown promise in clearing persistent methicillin-sensitive *Staphylococcus aureus* (MSSA) bacteraemia but has not been widely reported in oncology populations.

**Case summary:**

We present a case of 49-year-old woman with stage IA triple-negative breast cancer on chemotherapy via Port-a-Cath who was admitted to hospital with sepsis. Blood cultures revealed MSSA and echocardiography confirmed mitral valve vegetation, consistent with IE. Despite cefazolin therapy and port removal, bacteraemia persisted for 7 days. MRI and CT imaging revealed multiple embolic events, including cerebral infarcts and pulmonary emboli. Due to her poor surgical candidacy, combination therapy with IV nafcillin and ceftaroline was initiated. Fevers resolved and blood cultures cleared within 72 h. Ceftaroline was discontinued after 7 days, and nafcillin continued for 17 days before transitioning to cefazolin. She successfully completed 6 weeks of antibiotics with no recurrence of bacteraemia or IE.

**Discussion:**

This case demonstrates successful eradication of persistent MSSA IE using combination nafcillin and ceftaroline in a high-risk cancer patient for whom surgery was declined. It supports the potential role of dual beta-lactam therapy in managing complex IE when surgical options are limited.

Learning points

- Cancer patients are exposed to significant risks for bacteria and have greater rates of complications including infective endocarditis and septic emboli.
- Combination beta-lactam therapy can be an effective in clearing persistent MSSA bacteraemia due to complementary engagement of penicillin-binding domains.
- These combinations including nafcillin plus ceftaroline may be viable options for treating patients with infective endocarditis who are unable to tolerate surgical valve replacement.

## Introduction

Infective endocarditis (IE) is a dangerous sequela of bacteraemia that has a high rate of mortality without prompt and effective treatment with up to 30% mortality in all cases.^1^ Frequent vascular access and intravascular catheters represent common sources of bloodstream infections, and healthcare-associated infective endocarditis accounts for around half of all cases.^2^ Cancer patients undergoing chemotherapy via implanted venous access are at increased risk of bacteraemia with an infection rate of 0.04 per 100 days of catherization.^3^ Surgical intervention to remove and replace infected heart valves is indicated for bacteraemia that persists for 5–7 days, left heart infections with certain species including *Staphylococcus aureus*, and embolic events despite antibiotic therapy.^4^ However many patients have comorbid conditions that increase the risk of surgery to unacceptable levels, and approximately 50% of patients who are offered a surgical intervention reject it due to the high risk of post-surgical mortality.^2^ Combining multiple beta-lactams has been demonstrated to synergistically clear persistent *Staphylococcus aureus* by saturating a broad spectrum of penicillin-binding proteins (PBPs).^5^ Ceftaroline has been demonstrated to have a greater binding affinity for PBPs than previous generations of beta-lactams.^6^ Previous case series have shown combination therapy with nafcillin and ceftaroline to be successful in treating persistent methicillin-sensitive *Staphylococcus aureus* (MSSA) bacteraemia,^7^ but there are no reports of its use in cancer patients who are at greater risk for haematologic complications of bacteraemia including IE and septic emboli.^8^ Here we present a case of MSSA bacteraemia and IE with clear indications for surgery that resolved following nafcillin and ceftaroline combination therapy in a patient who had been receiving cancer chemotherapy via an implanted central venous catheter.

## Summary figure

**Figure:**
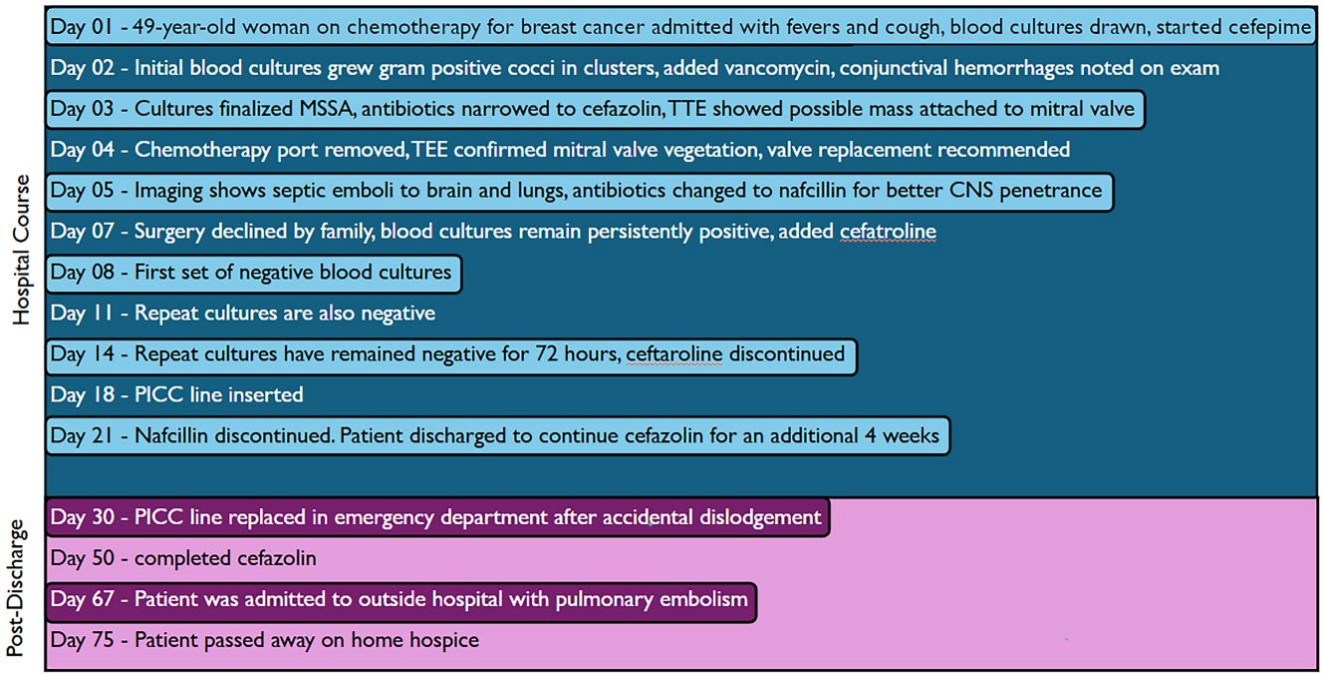


## Case presentation

A 49-year-old woman with history of developmental delays on active treatment for stage IA triple-negative breast cancer with adjuvant docetaxel and cyclophosphamide after partial mastectomy was admitted to the hospital for sepsis. She complained of fevers and cough over the past 4–5 days with watery, red eyes developing over the past 24 h. Earlier in the day she had come to the infusion centre with her mother, who was also her legal guardian, for her next dose of chemotherapy, and was sent to the emergency department for further evaluation. At the time of admission, her vital signs were a blood pressure of 129/76 mmHg, heart rate of 129 bpm, respiratory rate of 18 bpm, temperature of 38.1°C, and oxygen saturation of 96% on room air. On physical exam, she was alert and oriented with a thin body habitus. Her pupils were equal, round, and reactive to light with conjunctival injection and watery discharge from her eyes. Oral mucosa was dry and cracked. Her heart sounds were tachycardic in a regular rhythm, and no murmur was auscultated. Lungs were clear to auscultation bilaterally. She had a Port-a-Cath placed on the left-side of her chest and there was no oedema, erythema, or purulent drainage from the insertion site. Her abdomen was soft, non-distended, and non-tender. She did not have any skin lesions. Neurologic exam did not reveal any focal deficits, but she did have profound generalized weakness and debility. Laboratory results were significant for a neutrophilic leucocytosis with an elevated white blood cell count of 14.7k cells per cubic millimetre and absolute neutrophil count of 13.9k cells per cubic millimetre, and macrocytic anaemia with a haemoglobin of 10.2 g/dL and mean corpuscle volume of 103 fL. Viral respiratory polymerase chain reaction panel was negative for common pathogens including influenza and coronavirus. Other laboratory values including serum electrolytes, renal and hepatic function tests were unremarkable. EKG showed sinus tachycardia without evidence of ischaemia. Chest x-ray was clear. Urinalysis was not concerning for urinary tract infection.

**Figure 1:**
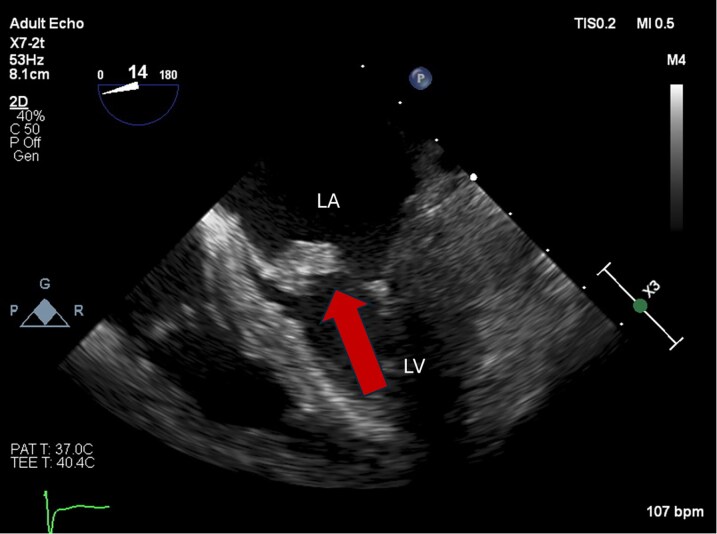
Transesophageal echocardiogram showing mitral valve vegetation.

**Figure 2:**
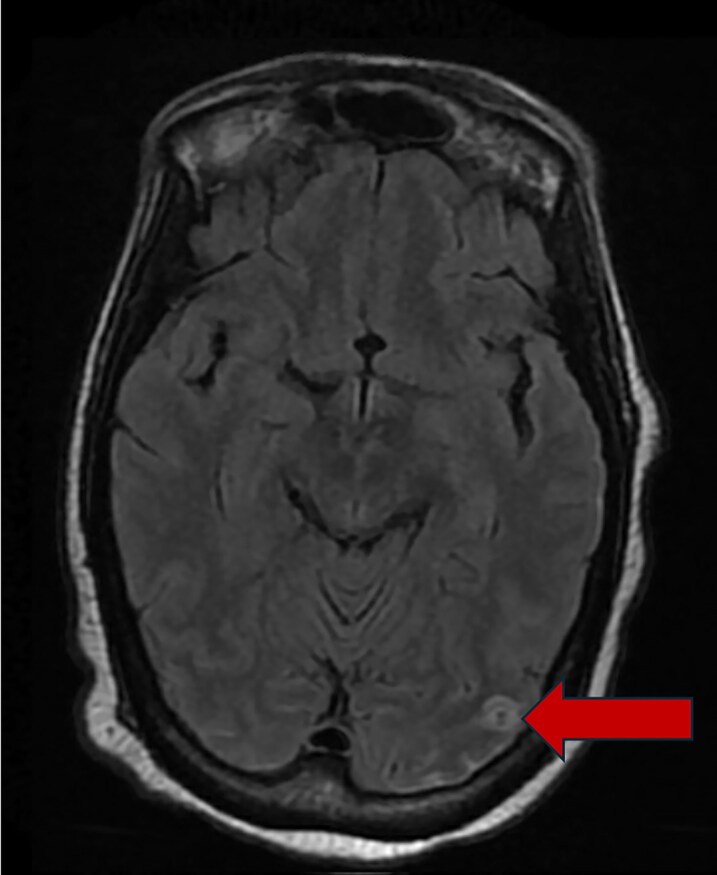
MRI brain showing left occipital lesion.

**Figure 3:**
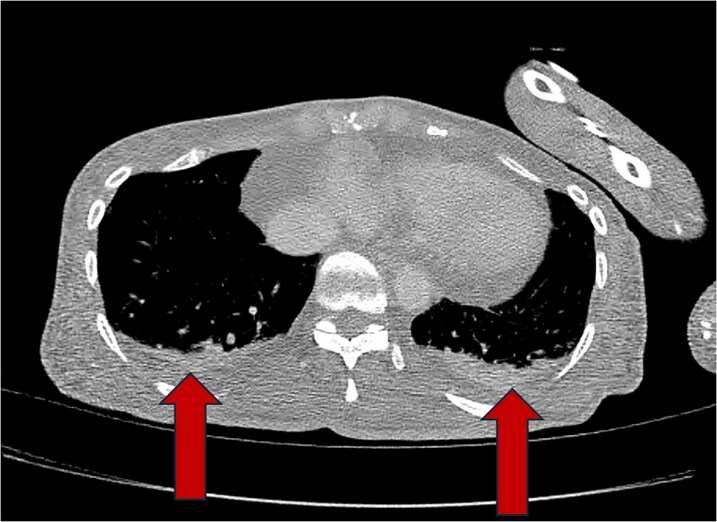
CT thorax showing bilateral pleural effusions and associated atelectasis/consolidation.

After drawing blood cultures, cefepime 2 g IV every 8 h was started for empiric treatment of suspected bacterial infection. Her initial blood cultures results showed gram positive cocci in clusters and vancomycin was added to her antibiotics with dosing managed by pharmacy according to blood concentrations. On further examination, she had small conjunctival haemorrhages in both eyes which were concerning for septic embolism from infective endocarditis. Transthoracic echocardiogram was performed which demonstrated a possible mobile mass attached to the anterior leaflet of the mitral valve. Final blood culture results MSSA, and antibiotic therapy was narrowed to cefazolin 2 g IV every 8 h. The chemotherapy port was considered to be the most likely source of infection and was removed by interventional radiology. Transoesophageal echocardiogram (*Figure 1*) confirmed the presence of a 0.5 × 0.5 cm vegetation attached to the atrial side of the anterior leaflet of the mitral valve. It also showed no mitral regurgitation, thickening of leaflets without impaired motion, and a mean pressure gradient across the mitral valve of 3.9 mmHg at a HR of 111 bpm suggestive of mild mitral stenosis Due to the concern for septic emboli, additional imaging was obtained to evaluate for seeding of other organ systems. Magnetic resonance imaging of the brain (*Figure 2*) showed multiple small foci of acute to subacute infarction in bilateral cerebellar hemispheres, left basal ganglia, left occipital lobe and corona radiata, and bilateral frontal cortex/subcortical white matter concerning for septic emboli, and computer tomography of the thorax (*Figure 3*) showed bilateral pleural effusions and consolidations concerning for embolic spread of her bloodstream infection. Due to presence of central nervous system involvement, antibiotics were switched to nafcillin 2 g IV every 4 h for better penetration of the blood–brain barrier. However, despite effective antibiotic therapy, she continued to be febrile and blood cultures persisted in growing MSSA over the first 7 days of admission.

Cardiothoracic surgery evaluated the patient for excision and replacement of the infected mitral valve. Surgical intervention was indicated due to left-sided infection with *Staphylococcus aureus* with persistently positive blood cultures and continued embolic events on an effective antibiotic regimen. However, the patient’s mother/legal guardian was concerned about the high risk of complications from an invasive procedure due to the patient’s poor performance status, and she declined to proceed with the recommended surgery. After discussion with family and infectious disease department, ceftaroline 600 mg IV every 12 h was added in combination with nafcillin. After initiating combination therapy, her fevers resolved and repeat blood cultures remained negative throughout admission. After repeat blood cultures had remained negative for 72 h, ceftaroline was discontinued after a total of 7 days of combination treatment. On day 21 of admission, nafcillin was discontinued after 17 days of treatment, and she was discharged to a skilled nursing facility with peripherally inserted central catheter (PICC) in place to continue cefazolin 2 g every eight hours for an additional 4 weeks due to the concern for renal toxicity with extended infusion of nafcillin. On day 30 after initial admission, she returned to the emergency department for replacement of her PICC line after it had been accidentally displaced. She completed the prescribed course of antibiotics and did not have any recurrence of MSSA bacteraemia or IE following discharge. On follow up, it was found that patient had been admitted to an outside hospital due to pulmonary embolism likely provoked by her cancer and had passed away on day 75 after initial admission.

## Discussion

This case illustrates a successful use of combination therapy with nafcillin and ceftaroline in a patient with MSSA infective endocarditis who was a poor surgical candidate for valve vegetation removal. Following successful antibiotic treatment for endocarditis, most patients have persistent vegetations. Unfortunately, in this case, a follow-up echocardiogram could not be performed after completion of antibiotics prior to the patient passing away to ensure continued adequate mitral valve function. There are limited reports in literature on the successful outcomes of this and other combinations of beta-lactams in treating persistent MSSA infections. Cefazolin is typically the preferred agent for treatment of uncomplicated MSSA bacteraemia due to a lower risk of mortality,^9^ and a review of previous case series have suggested that combination of an anti-staphylococcal beta-lactam with ertapenem may be a superior regimen in treating persistent MSSA infections.^10^ However, in this case, cefazolin was discontinued in favour of nafcillin due to the discovery of brain lesions consistent with septic emboli as nafcillin achieves greater concentrations in the central nervous system than cefazolin.^11^ Additionally, combination with ceftaroline allowed the avoidance of ertapenem in a time where carbopenem resistance rates continue to rise.^12^ Combination therapy with daptomycin or gentamycin may also have been able to achieve a similar result.^10^ Additional investigation is needed into the use of combination antibiotic therapy for treatment of MSSA IE in settings where surgical intervention is not possible, and the combination of nafcillin plus cefazoline may represent a viable option particularly in cases where there is also central nervous system involvement. Such investigations would especially benefit cancer patients who are at a high risk of developing complications from blood stream infections and often have comorbidities that make it desirable to avoid surgery.

## Lead author biography


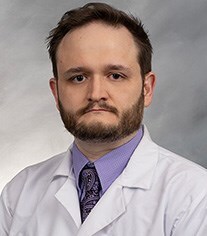


Joshua Modrick is an internal medicine resident currently in training at Wayne State University/Detroit Medical Center. He earned his Bachelor’s in Science from the University of Iowa and his Master’s in Science from Johns Hopkins University before attending medical school at Des Moines University College of Osteopathic Medicine. He hopes to pursue fellowship in Hematology/Oncology.

## Data Availability

All data are incorporated into the article.

## Notes

**Consent:** Informed consent for publication of this article was obtained from the patient’s legal guardian in accordance with COPE guidelines.
